# Quantitative and qualitative image quality assessment in shoulder examinations with a first-generation photon-counting detector CT

**DOI:** 10.1038/s41598-023-35367-2

**Published:** 2023-05-22

**Authors:** Theresa Sophie Patzer, Andreas Steven Kunz, Henner Huflage, Karsten Sebastian Luetkens, Nora Conrads, Philipp Gruschwitz, Pauline Pannenbecker, Süleyman Ergün, Thorsten Alexander Bley, Jan-Peter Grunz

**Affiliations:** 1 Department of Diagnostic and Interventional Radiology, University Hospital Würzburg, Oberdürrbacher Str. 6, 97080 Würzburg, Germany; 2 Institute of Anatomy and Cell Biology, University of Würzburg, Koellikerstr. 6, 97070 Würzburg, Germany

**Keywords:** Musculoskeletal system, Bone

## Abstract

Photon-counting detector (PCD) CT allows for ultra-high-resolution (UHR) examinations of the shoulder without requiring an additional post-patient comb filter to narrow the detector aperture. This study was designed to compare the PCD performance with a high-end energy-integrating detector (EID) CT. Sixteen cadaveric shoulders were examined with both scanners using dose-matched 120 kVp acquisition protocols (low-dose/full-dose: CTDI_vol_ = 5.0/10.0 mGy). Specimens were scanned in UHR mode with the PCD-CT, whereas EID-CT examinations were conducted in accordance with the clinical standard as “non-UHR”. Reconstruction of EID data employed the sharpest kernel available for standard-resolution scans (ρ_50_ = 12.3 lp/cm), while PCD data were reconstructed with both a comparable kernel (11.8 lp/cm) and a sharper dedicated bone kernel (16.5 lp/cm). Six radiologists with 2–9 years of experience in musculoskeletal imaging rated image quality subjectively. Interrater agreement was analyzed by calculation of the intraclass correlation coefficient in a two-way random effects model. Quantitative analyses comprised noise recording and calculating signal-to-noise ratios based on attenuation measurements in bone and soft tissue. Subjective image quality was higher in UHR-PCD-CT than in EID-CT and non-UHR-PCD-CT datasets (all p < 0.001). While low-dose UHR-PCD-CT was considered superior to full-dose non-UHR studies on either scanner (all p < 0.001), ratings of low-dose non-UHR-PCD-CT and full-dose EID-CT examinations did not differ (p > 0.99). Interrater reliability was moderate, indicated by a single measures intraclass correlation coefficient of 0.66 (95% confidence interval: 0.58–0.73; p < 0.001). Image noise was lowest and signal-to-noise ratios were highest in non-UHR-PCD-CT reconstructions at either dose level (p < 0.001). This investigation demonstrates that superior depiction of trabecular microstructure and considerable denoising can be realized without additional radiation dose by employing a PCD for shoulder CT imaging. Allowing for UHR scans without dose penalty, PCD-CT appears as a promising alternative to EID-CT for shoulder trauma assessment in clinical routine.

## Introduction

With its large range of motion and resulting susceptibility to instability, the glenohumeral articulation represents a highly vulnerable joint for trauma-associated dislocation. Beside soft tissue injuries of the rotator cuff, fractures play a major role as concomitant injuries. For instance, glenoid fractures represent the most common accompanying injury in anterior shoulder dislocation^1^. Due to the variety of trauma mechanisms and forces of impact, suchlike fractures range from subtle fissures to dislocated multi-fragment injuries. With its cost efficiency and ubiquitous availability at relatively low radiation dose, digital radiography remains the primary imaging method for fracture diagnosis. Regardless of the higher radiation burden inherent to computed tomography (CT), ancillary cross-sectional examinations are mandatory for detailed display of bone microarchitecture, to ascertain radiographically occult fractures, and for preoperative evaluation of fracture morphology^2,3^. Thus far, energy-integrating detector (EID) CT systems have represented the clinical standard for 3D examinations of the appendicular skeleton in the context of trauma. Typical limitations include the trade-off between limited spatial resolution in regular scan mode and dose penalties in ultra-high-resolution mode (UHR), while partial volume effects and image noise hamper the dedicated depiction of fine bone structures especially in low-dose settings and when scanning obese patients^4^.

The dose-saving potential of EID-CT systems is limited due to increased electronic noise and energy-weighting, with low-energy photons contributing less to the overall detector signal^5^. Providing very high spatial resolution up to 0.11 mm in UHR mode, photon-counting detector (PCD) CT systems offer the option to address these challenges. In contrast to standard EID-CT, where geometric dose efficiency is encumbered by constructional restrictions such as optical separation layers, PCD require no such septa. Instead, detector pixels are characterized by an electric field and a pixelated anode, thus allowing for increased spatial resolution by separate readout of smaller subpixels^6,7^. Opposed to EID, which employ scintillator elements, PCD contain semiconductors (e.g., cadmium telluride) enabling photons to be directly converted into a proportional electrical signal, rendering the additional step of transformation into visible light unnecessary^8,9^.

In EID-CT, the total amount of energy conveyed by the entirety of photons is merged, including electronic noise. In contrast, PCD register electrical pulses generated by every single photon reaching the detector when they exceed a predefined threshold. If this threshold is maintained higher than the level of electronic noise, low-level image noise does not contribute to the count rate^10^. While an increasing number of publications confirm the technology’s potential^11–13^, PCD’s capability for visualizing trabecular bone microstructure in large joints has not been evaluated based on radiation-dose equivalent comparisons with an EID-CT system thus far.

This study was designed to compare quantitative and qualitative image quality parameters between a first-generation PCD-CT scanner and a high-end dual-source EID-CT in dose-matched shoulder imaging. We hypothesized that the novel detector technology would allow for substantially better image quality, thus offering potential for considerable dose saving.

## Material and methods

This investigation and all experimental protocols were granted approval by the Institutional Review Board of the University of Würzburg, Germany. All experiments were performed in accordance with relevant guidelines and regulations. Eight formalin-fixed cadaveric bodies were obtained from the Institute of Anatomy and Cell Biology of the University of Würzburg, Germany. During their lifetime, donors volunteered their corpses to this institution for study and research purposes, hence additional written informed consent was not required.

### Image acquisition and reconstruction

Examinations of 16 cadaveric shoulders were executed on a clinical first-generation PCD-CT system (Naeotom Alpha; Siemens Healthcare GmbH, Forchheim, Germany) and a third-generation dual-source CT scanner with an energy-integrating detector (Somatom Force; Siemens Healthcare GmbH). Employing the EID-CT system, cadaveric shoulder scans were carried out in the clinically established single-energy mode. To ensure dose-matched comparisons between the two detector technologies, EID-CT scans were conducted in non-UHR mode, since UHR collimation is subjoined with an increased radiation burden due to the necessity of adding a post-patient comb filter to narrow the detector aperture. Irrespective of scanner type, studies were conducted with a helical pitch factor of 0.5. Detector collimation was 96 × 0.6 mm in EID-CT and 120 × 0.2 mm in PCD-CT. Two EID-CT acquisition protocols were employed with a fixed 120 kVp tube voltage and varying tube current–time products (74 and 149 effective mAs) resulting in volume CT dose indices (CTDI_vol_) of 5.0/10.0 mGy (low-dose/full-dose), respectively. Maintaining a tube voltage of 120 kVp, acquisition protocols for PCD-CT scans were designed to equal the CTDI_vol_ of scan protocols for their EID-CT counterparts, resulting in tube current–time products of 62 and 126 effective mAs. Image acquisition settings are summarized in Table 1.

**Table 1:** Acquisition settings.

Acquisition parameter	PCD-CT		EID-CT	
	Full-dose	Low-dose	Full-dose	Low-dose
Tube potential [kVp]	120	120	120	120
Tube current [mAs]	126	62	149	74
Collimation [mm]	120 × 0.2	120 × 0.2	96 × 0.6	96 × 0.6
Pitch factor	0.5	0.5	0.5	0.5
Rotation time [sec]	1.0	1.0	1.0	1.0
CTDI_vol_ [mGy]	10.0	5.0	10.0	5.0

*CTDI*_*vol*_ volume computed tomography dose index, *EID-CT* energy-integrating CT, *PCD-CT* photon-counting CT.

To guarantee identical in-plane resolution irrespective of system type and acquisition parameters, reformatting was conducted in standard planes with an increment of 0.5 mm and a slice thickness of 1 mm. Reconstruction of all acquired data was executed with a field of view of 120 mm and a 512 × 512 pixel matrix. For optimized evaluation of bone structure, window settings were predefined to 1500/450 HU (width/center), though readers were allowed to adjust the contrast during image analysis. As bone structure assessment represented the primary goal of this investigation, the sharpest kernel available for non-UHR scans (Br69, Siemens; ρ_50_ = 12.3 line pairs per cm [lp/cm]; ρ_10_ = 15.1 lp/cm) was chosen for reconstruction of EID-CT data. Since convolution kernels are exclusive to one scanner type, it was not possible to use the exact same kernel for reconstruction of EID-CT and PCD-CT datasets. Therefore, two different convolution kernels were selected for reformatting of PCD-CT raw data: A non-UHR kernel (Br68, Siemens; ρ_50_ = 11.8 lp/cm; ρ_10_ = 14.5 lp/cm) that matches the modulation transfer function (MTF) of EID-CT reconstructions as close as technically possible, and a sharper bone kernel characterized by a higher spatial frequency (Br76, Siemens; ρ_50_ = 16.5 lp/cm, ρ_10_ = 21.0 lp/cm). Of note, though, the cadmium-telluride-based PCD facilitates a maximum spatial resolution greater than either of the chosen kernels.

Due to exclusive iterative reconstruction algorithms for each detector technology, reformatting with varying settings was unavoidable. EID-CT raw data were reconstructed with a third-generation algorithm (strength level 3; ADMIRE, Siemens Healthcare GmbH), whereas PCD-CT data reconstruction was carried out with a fourth-generation algorithm (strength level 2; QIR, Siemens Healthcare GmbH). Notably, performing reconstructions with sharp bone kernels and ADMIRE strength level 3 is comparable to QIR strength level 2, according to vendor information. However, it must be taken into account that by employing the PCD-CT scanner’s iterative reconstruction algorithm, denoising is performed automatically on the basis of a 20 keV energy threshold^5^.

### Subjective image analysis

Subjective image analysis was carried out in standardized settings using clinical picture archiving and communication software (Merlin; Phönix-PACS, Freiburg, Germany) in combination with monitors certified for diagnostic use (RadiForce RX660; EIZO, Hakusan, Japan). All datasets were independently evaluated by six radiologists with varying expertise in skeletal imaging (ranging between 2 and 9 years). Based on an equidistant seven-point scale, readers were asked to rate image quality of cancellous and cortical bone(= excellent; 6 = very good; 5 = good; 4 = satisfactory; 3 = fair; 2 = poor; 1 = very poor). No time limit was imposed and observers received no scan protocol-related information for their reads.

### Objective image analysis

**Figure 1:**
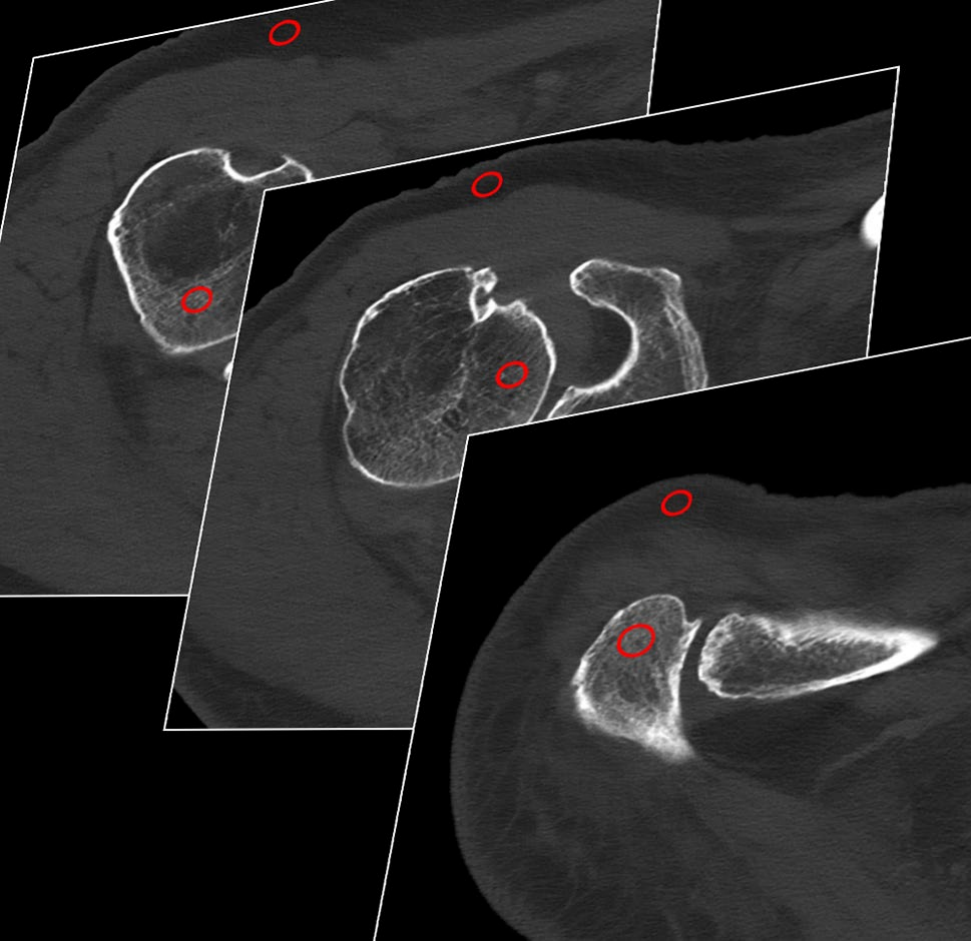
CT number measurements within regions of interest placed in the cancellous bone of the shoulder as well as in adjacent subcutaneous fat tissue on three consecutive axial CT slices.

A radiologist with two years of experience in musculoskeletal evaluation performed objective image analysis using specific software (syngo.via VB60A; Siemens Healthcare GmbH). Image noise was recorded based on CT number measurements on axial reconstructions, manually placing standardized regions of interest (ROIs) in predefined, concordant positions within cancellous bone of the glenoid, acromion, and humeral head, as well as in circumjacent subcutaneous fat tissue. ROIs size was preset to 100 mm^2^, however, to avoid measurement of unrepresentative tissue size could be reduced. Within each technique (i.e., all PCD-CT data/all EID-CT data), ROIs were copied between datasets, since no repositioning of specimens took place between scans on different dose levels. For each ROI, mean signal attenuation and standard deviation were measured in Hounsfield units (HU) in order to compute signal-to-noise ratios (SNR). To ensure high measurement accuracy and data consistency, ROIs were placed on three sequential axial CT slices of each shoulder with averaging of measurements (Fig. 1). In contrast to cancellous bone, adipose tissue provides a more homogenous texture, thus defining noise as HU standard deviation within subcutaneous fat tissue^14^.

### Data analysis

Dedicated software (SPSS Statistics 28, IBM, Armonk, USA) was used for statistical analysis. Reporting of ordinal data comprises median values and interquartile ranges (IQR), whereby normally distributed continuous variables are reported as means ± standard deviations. Comparison of absolute noise levels and signal-to-noise ratios was performed with one-way analyses of variance (ANOVA) and Bonferroni-corrected pairwise post-hoc tests. Similarly, Friedman’s ANOVA and pairwise post-hoc comparisons were performed for non-parametric variables. Significance is indicated by an alpha level of 0.05.

## Results

### Subjective image analysis

**Figure 2:**
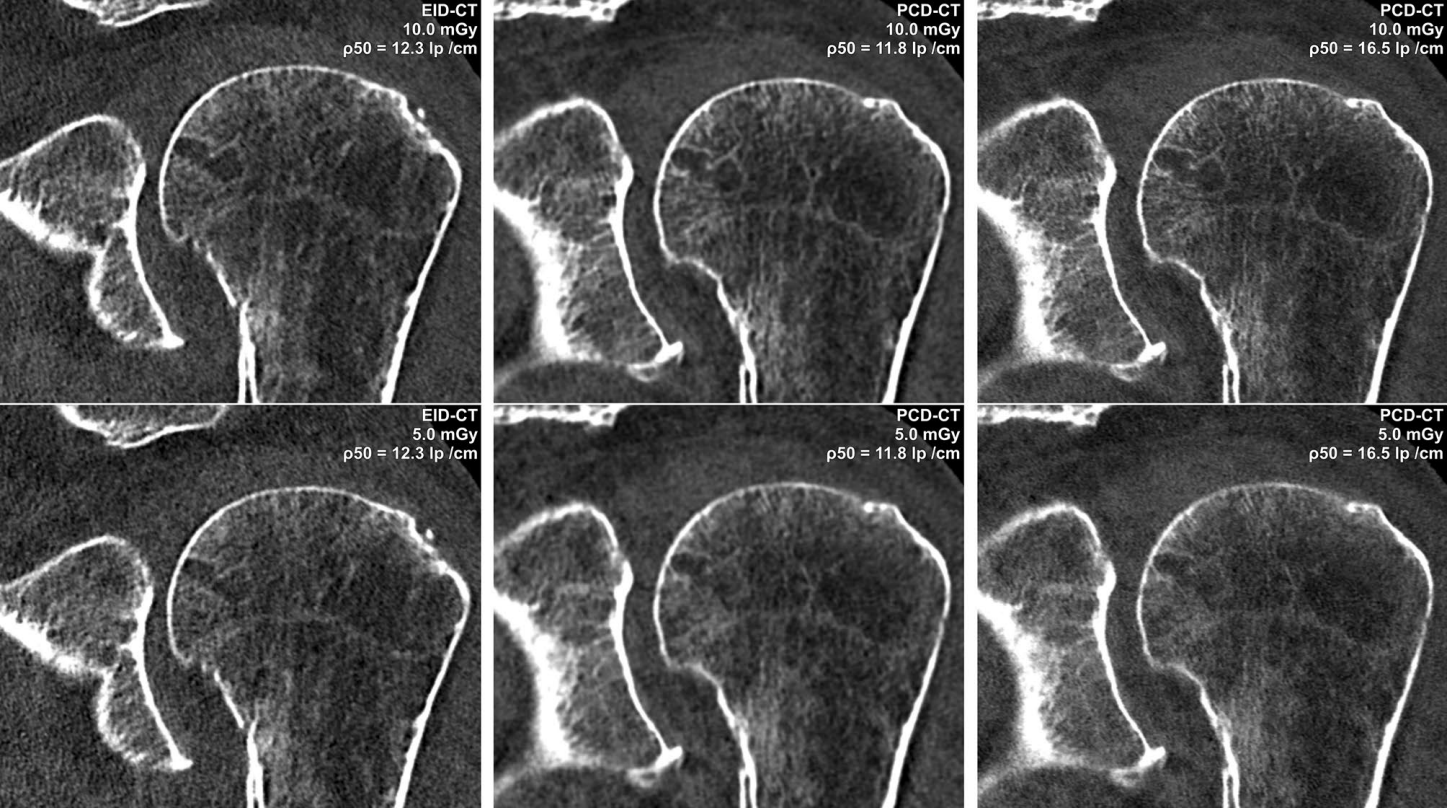
Left column: Coronal reconstruction of standard-resolution shoulder CT with the sharpest possible kernel (ρ_50_ = 12.3 lp/cm) and two dose levels on a third-generation dual-source EID-CT. *Middle column* Ultra-high-resolution PCD-CT examinations with a comparable convolution kernel (ρ_50_ = 16.5 lp/cm) allow for considerable noise reduction over dose-matched EID-CT scans. Right column: Superior delineation of bone microarchitecture can be achieved when employing dedicated high-resolution kernels (ρ_50_ = 11.8 lp/cm) even with decreased radiation dose.

Image quality ratings were highest for full-dose UHR-PCD-CT (median value 7, [IQR 6–7]). Irrespective of dose level, image quality of UHR-PCD-CT datasets was considered preferable to EID-CT and non-UHR-PCD-CT reconstructions (all p < 0.001). Figure 2 includes representative coronal CT slices of each dataset in collated fashion. Readers even attributed superior image quality to low-dose UHR-PCD-CT reconstructions (6 [5, 6]) compared to full-dose non-UHR-PCD-CT (5 [5]) and full-dose EID-CT datasets (4 [4, 5]). While the image quality of non-UHR-PCD-CT was deemed preferable to EID-CT on either dose level, no difference was ascertained between low-dose non-UHR-PCD-CT and full-dose EID-CT (both 4 [4, 5]); p > 0.99). Image quality ratings of the six radiologists for each combination of scanner, radiation dose, and image reconstruction are summarized in Table 2. Indicated by a single-measure intraclass correlation coefficient of 0.66 (95% confidence interval: 0.58–0.73; p < 0.001), inter-reader reliability was moderate.

**Table 2:** Subjective image quality.

	UHR-PCD-CT		Non-UHR-PCD-CT		EID-CT	
	Full-dose	Low-dose	Full-dose	Low-dose	Full-dose	Low-dose
7	52 (54.2%)	14 (14.6%)	2 (2.1%)	0	0	0
6	35 (36.5%)	48 (50.0%)	17 (17.7%)	8 (8.3%)	10 (10.4%)	3 (3.1%)
5	9 (9.4%)	34 (35.4%)	56 (58.3%)	34 (35.4%)	31 (32.3%)	6 (6.3%)
4	0	0	21 (21.9%)	45 (46.9%)	48 (50.0%)	27 (28.1%)
3	0	0	0	9 (9.4%)	7 (7.3%)	47 (49.0%)
2	0	0	0	0	0	13 (13.5%)
1	0	0	0	0	0	0
Median (IQR)	7 (6–7)	6 (5–6)	5 (5–5)	4 (4–5)	4 (4–5)	3 (3–4)

Pooled image quality ratings of six radiologists for PCD- and EID-CT examinations in 16 shoulders of cadaveric specimens (7 = excellent; 6 = very good; 5 = good; 4 = satisfactory; 3 = fair; 2 = poor; 1 = very poor).

*EID-CT* energy-integrating CT, *IQR* interquartile range, *PCD-CT* photon-counting CT, *UHR* ultra-high resolution.

### Objective image analysis

**Figure 3:**
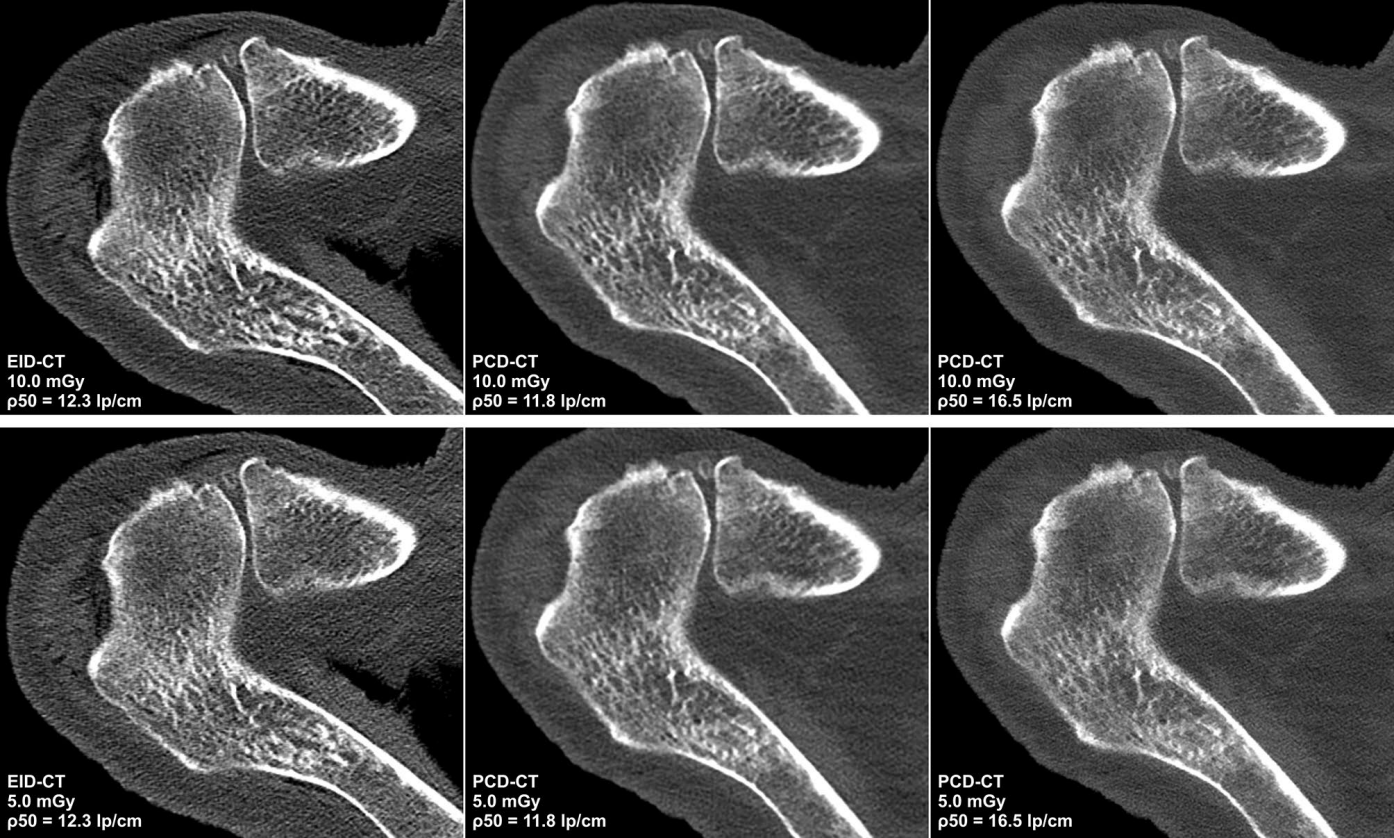
Representative axial CT slices at the level of the acromioclavicular joint demonstrate the image quality with all six combinations of dose protocol and detector technology. Increased image noise with lower dose in EID-CT limits assessability of cancellous bone structures. Upper row: Full-dose scan protocols (10.0 mGy): EID-CT, non-UHR-PCD-CT, UHR-PCD-CT. Lower row: Low-dose scan protocols (5.0 mGy): EID-CT, non-UHR-PCD-CT, UHR-PCD-CT.

The lowest image noise overall was recorded for full-dose non-UHR-PCD-CT (40.3 ± 7.2 HU), corresponding to the highest SNR among all scan protocols (4.2 ± 1.5). In contrast, the highest image noise was measured in low-dose EID-CT (97.7 ± 18.0 HU), corresponding with the lowest SNR (1.8 ± 0.7). Side-by-side analysis of axial reformations of EID-CT and PCD-CT data used for ROI-based measurements is provided in Fig. 3. Comparing the individual PCD-CT datasets, image noise was lowest and SNR were highest in non-UHR reconstructions at either dose level. Notably, no difference was found between full-dose UHR (noise: 48.9 ± 7.5 HU; SNR: 3.5 ± 1.2) and low-dose non-UHR-PCD-CT (48.4 ± 7.7 HU; 3.5 ± 1.3) for both objective criteria assessed within this study (both p > 0.99). Nevertheless, UHR-PCD-CT reconstructions at either dose level were associated with considerably less image noise than the dose-equivalent EID-CT datasets. Even between full-dose EID-CT (noise: 78.2 ± 6.4 HU; SNR: 2.2 ± 0.9) and low-dose UHR-PCD-CT (48.4 ± 7.7 HU; 3.5 ± 1.3), superior results regarding the latter were ascertained (both p < 0.001). Quantitative criteria of image quality for each scan and reconstruction are summarized in Table 3.

**Table 3:** Objective image quality.

	UHR-PCD-CT		Non-UHR-PCD-CT		EID-CT	
	Full-dose	Low-dose	Full-dose	Low-dose	Full-dose	Low-dose
Image noise [HU]	48.9 ± 7.5	58.6 ± 7.4	40.3 ± 7.2	48.4 ± 7.7	78.2 ± 16.4	97.7 ± 8.0
Signal-to-noise ratio	3.5 ± 1.2	2.9 ± 1.0	4.2 ± 1.5	3.5 ± 1.3	2.2 ± 0.9	1.8 ± 0.7

Signal-to-noise ratios were calculated based on Hounsfield unit measurements in consistent locations within the cancellous bone of the shoulder and adjacent subcutaneous fat. Results are displayed as mean values ± standard deviations.

*EID-CT* energy-integrating CT, *PCD-CT* photon-counting CT, *UHR* ultra-high resolution.

## Discussion

This study was designed to investigate the performance of two top of the line CT scanners, comparing a dual-source system equipped with energy-integrating detector technology and a cadmium-telluride-based photon-counting detector CT system. In radiation dose-matched examinations of 16 cadaveric shoulders, subjective image analysis revealed superior ratings for UHR-PCD-CT over standard-resolution reconstructions of scans acquired with either detector technology, irrespective of dose level. Notably, image noise was lowest and SNR was highest in datasets reconstructed below the UHR capabilities of the PCD at either dose level.

Multiple studies have reported the capability of relevant image noise reduction in PCD-CT systems^15–17^. Studies investigating the PCD-CT system’s potential for trabecular bone microstructure visualization in phantoms, as well as in vivo, e.g., for paranasal sinus, temporal bone, peripheral joint, and pelvis imaging have shown promising results regarding the detailed depiction of cancellous bone^18–26^. While precise display of bone microarchitecture is mandatory for detection of subtle fissures and adequate preoperative evaluation of fracture morphology in shoulder examinations, the primary approach to provide high spatial resolution is scanning in UHR mode. Even though both scanner systems are technically capable of UHR imaging, conducting EID-CT scans in UHR mode implicates the utilization of an additional post-patient comb filter to narrow the detector aperture. Hence, UHR-EID-CT is associated with a restricted field of view (approx. 35 cm), as well as reduced dose efficiency and consequently increased noise levels^25^. Whereas EID-CT with UHR collimation is still applied for depiction of extremities and the temporal bone region, filter-based UHR-EID-CT is not established for body trunk examinations due to considerably increased image noise or radiation dose^26^. It should also be stated that the manufacturer does not provide UHR-EID-CT protocols for the shoulder, hence detailed display of fine trabecular anatomy is naturally restricted for this anatomical location when using EID scanner architecture. Contrarily, employing UHR mode in PCD-CT scans is not associated with a higher radiation burden, allowing for considerable dose reduction over conventional EID-CT^7,11^. Since we aimed to compare realistic scan protocols that could be applied in clinical routine, we refrained from including any self-customized UHR EID-CT shoulder protocols.

Reconstruction with sharper convolution kernels represents another method to improve delineation of bone microarchitecture. In order to account for exclusive technical parameters inherent to both detector technologies, PCD-CT data reconstruction was performed with a convolutional kernel matching the MTF of the sharpest kernel available for optimal bone visualization employing EID-CT in standard-resolution mode. To allow for higher spatial resolution, PCD-CT data reconstruction was additionally carried out with a sharper bone kernel, providing superior subjective image quality compared to EID-CT and non-UHR PCD-CT reconstructions, irrespective of dose level. Nevertheless, it needs to be considered that image sharpness achieved with these kernels remains considerably lower than the raw MTF for PCD-CT in UHR mode. Even though the Br68 kernel does not fully exploit the PCD’s capability of high-resolution bone depiction at this time, superior denoising and highest SNR were recorded on either dose level. Potentially explaining the observers’ distinct preference of UHR-PCD-CT reconstructions, the sharper kernel allows for superior differentiation of trabeculae, albeit at the cost of increased image noise. Concordant with literature^18^, PCD-CT reconstructions with either kernel were associated with substantially less image noise than EID-CT scans, though. Considering that low-dose UHR-PCD-CT was deemed superior to full-dose studies with standard reformatting on both scanners, our results support the hypothesis that reconstruction with a sharper kernel offers the potential for substantial dose reduction^13^.

In contrast to our results, image noise did not differ significantly in the investigation by Baffour et al.^21^, evaluating clinical shoulder scans in state-of-the-art EID-CT and PCD-CT. This finding is most likely attributed to the significant dose reduction in this study (47% lower CTDI_vol_ in PCD-CT compared to EID-CT) with consequently comparable level of image noise. Even though their study revealed a remarkable radiation reduction, they were able to achieve superior depiction of fine bone structures in PCD-CT scans. However, dose values in our investigation were considerably below the settings evaluated by Baffour et al. (PCD 18 mGy/EID 33.8 mGy vs. 5 mGy/10 mGy in our study). Considering the ALARA principle (as low as reasonably achievable), dose levels were chosen in accordance to the German diagnostic reference levels (DRLs) with 10 mGy representing the upper limit for CT investigations of the thorax^27^. Comparing two high-end CT systems, it needs to be acknowledged that most scanners currently available are not capable of achieving similarly low radiation levels as applied in this study with comparable image quality.

Some limitations of this study have to be discussed. First, only eight cadaveric specimens with a total of 16 shoulders were investigated. Information regarding the duration of formalin fixation, as well as the body donors’ age and bone density remained unknown. Irrespective of scanner and scan protocol, the heterogenous extent of bone demineralization may have hampered image quality assessment^28,29^. Second, as dedicated visualization of bone represented the primary aim, soft tissue analysis was not the scope of this study. Third, acquisition parameters and reconstruction kernels were selected matching the clinical standard in EID-CT scans. Thus, the potential of PCD scanners is possibly underestimated in our current study. Fourth, deriving the impact of detailed depiction of bone microarchitecture in a real-world population from image quality analysis in cadaveric shoulder examinations requires some extrapolation. Consequently, further studies are warranted to compare scanner performance in clinical practice.

## Conclusion

Superior depiction of fine trabecular structures and considerable denoising can be realized with PCD-CT in cadaveric shoulder scans. Particularly, the option to perform UHR scans without an added dose penalty suggests potential for decreasing the radiation exposure over conventional EID-CT.

## Data Availability

The datasets generated and/or analyzed during the current study are not publicly available since participants of this study did not agree to their data being shared in a public repository. However, datasets are available from the corresponding author on reasonable request.

## Abbreviations

CTDI_vol_: Volume computed tomography dose index
EID: Energy-integrating detector
IQR: Interquartile range
PCD: Photon-counting detector
ROI: Region of interest
SNR: Signal-to-noise ratio
UHR: Ultra-high-resolution
